# Comparison of Mid-Upper Arm Circumference and Weight-for-Height to Diagnose Severe Acute Malnutrition: A Study in Southern Ethiopia

**DOI:** 10.3390/nu9030267

**Published:** 2017-03-11

**Authors:** Amare Worku Tadesse, Elazar Tadesse, Yemane Berhane, Eva-Charlotte Ekström

**Affiliations:** 1 Department of Women’s and Children’s Health, International Maternal and Child Health Uppsala University, SE-75185 Uppsala, Sweden; elazar.balla@kbh.uu.se (E.T.); Lotta.Ekstrom@kbh.uu.se (E.-C.E.); 2 Department of Public Health Sciences, Addis Continental Institute of Public Health, P.O. Box 26751/1000 Addis Ababa, Ethiopia; yemaneberhane@gmail.com

**Keywords:** MUAC, WHZ, severe acute malnutrition, agreement, age, sex and children

## Abstract

Weight-for-height *Z*-score (WHZ) and mid-upper arm circumference (MUAC) are two independent anthropometric indicators for diagnosing and admitting children with severe acute malnutrition (SAM) for treatment. While severely wasted children are at high risk of mortality, MUAC and WHZ do not always identify the same population of children as having SAM. Understanding how this discrepancy relates to age and sex may provide valuable information for care programmes for children with SAM. Age and sex distribution for differences between children identified as SAM by MUAC and WHZ were examined and the degree of agreement calculated. Children (*n* = 4297) aged 6–59 months with validated anthropometric measures were recruited from a population-based survey conducted in rural southern Ethiopia. MUAC < 115 mm and WHZ < −3 were used to define severe wasting as per the World Health Organization (WHO) classification. The kappa coefficient (κ) was calculated. There was fair agreement between the MUAC and WHZ definitions of severe wasting in boys (κ = 0.37) and children younger than 24 months (κ = 0.32) but poor agreement in girls (κ = 0.15) and children aged 24 months and above (κ = 0.13). More research is needed on response to treatment and prediction of mortality using different anthropometric measurements in relation to ages and sex of children.

## 1. Introduction

Acute malnutrition considerably increases the risk of childhood morbidity and mortality, and is reported to be responsible for nearly 875,000 (13%) of the global deaths in children under five years of age [1]. Severe acute malnutrition (SAM) contributes to almost three-fifths of these deaths [1]. If identified and properly managed, it is possible to prevent these deaths [2]. Previously, the World Health Organization (WHO) guideline for SAM management was restricted to inpatient management of SAM [3]. Currently, the recommended standard management for all children with SAM is Community Management of Severe Acute Malnutrition (CMAM) [4,5].

While weight-for-height (WHZ) has long been the indicator of choice for diagnosing SAM in primary health care facilities, it has technical and practical limitations in its use by community health workers under field conditions. Thus, a two-stage referral and admission system evolved to simplify community-level operations. The community health workers identify and refer SAM children based on mid-upper arm circumference (MUAC), while programme staff admit SAM children to Outpatient Therapeutic Program (OTP) based on WHZ [6].

In contrast to WHZ, MUAC is a simple and low-cost method [4] that can be applied easily by one person after minimum training [6] and is less susceptible to measurement error than WHZ [7]. However, the two-stage referral procedure means that many children who have been referred based on MUAC are refused treatment as they do not meet the WHZ-based admission criteria. This problem was solved by also introducing MUAC as an alternative criterion for both referral and admission to OTP [6,8]. The use of MUAC for screening and admission can also be defended by its greater ability to predict the risk of mortality than WHZ [9,10,11]. Current guidelines recommend the use of MUAC < 115 mm or WHZ < −3 independently for the identification of severe wasting in children aged 6–59 months [12,13].

The WHO growth standards are reflective of how children worldwide have the potential to grow and develop as long as their basic needs are met, including nutrition, healthcare and environment [13]. To classify inappropriate child growth or nutritional status, a child’s growth is compared with the growth standard curve constructed from these healthy child populations commonly applying a cut-off value of <−2 or >+2 *Z*-scores. The rationale for these cut offs is the statistical definition of the central 95% of a distribution as the ‘normal’ range, which is not necessarily based on the optimal point for predicting risk of health outcomes. Similarly, the MUAC cut-off points were selected from statistical analysis of nutritional surveys to approximately correspond to the WHZ cut-off points so that the same prevalence of acute malnutrition would be found with each criterion [13].

While both indicators are commonly used in programmes providing care for SAM children, MUAC and WHZ have been shown to identify children as severely wasted inconsistently [13,14,15]. Furthermore, the discrepancy between the indicators is shown to vary in different settings [13,14,15]. However, the extent of overlap and discordance in relation to age and sex between the different children identified as severely wasted is not well defined. These observed differences often have programmatic implications [16]. If the diagnosis of severe wasting can be based on the use of either indicator [13,14], then it may unreasonably increase the health care cost and workload of programmes providing care for SAM, as the most appropriate management of children identified by one indicator and not by the other creates uncertainty. Alternatively, a shift to single MUAC criterion for admission to community-based therapeutic programmes may result in missed opportunities to treat a severe condition [14].

The primary aim of programmes providing care for SAM children is to prevent mortality, thus indicators used should identify children who are at high risk of dying. The relationship between low anthropometric status and increased mortality is widely accepted and the influence of age and sex on childhood mortality risk has been documented [10,11,17,18,19,20]. As MUAC and WHZ identify different populations of children as severely wasted, it would be helpful to know how this discrepancy between the indicators is related to difference in selection of children in terms of age or sex. This issue is important to malnourished children between the ages of 6 and 59 months, for whom there is uncertainty in the criteria for admission and discharge because of limited evidence to improve current treatment guidelines [12]. Thus, we aimed to describe the age and sex distribution of children diagnosed as severely wasted using MUAC and WHZ, and to assess the degree of agreement between these anthropometric indicators in children aged between 6 and 59 months from rural southern Ethiopia.

## 2. Methods

### 2.1. Study Setting and Design

A population-based survey (COMSAM) was conducted to evaluate the effectiveness of integrated CMAM in rural districts of southern Ethiopia. The study area mainly comprised mid- and lowland agro-ecologies. The districts practise crop-livestock mixed farming and keep a combination of livestock integrated with a wide range of cereals, pulses, roots and tubers and cash crops grown for household consumption and marketing. However, recurrent drought and subsequent crop failure in the area has resulted in cyclical nutritional emergencies and chronic food insecurity [21].

### 2.2. Participants

Out of twelve rural districts in the region, four adjacently situated districts and all of their rural kebeles (the smallest administrative unit in Ethiopia) were purposively selected as these districts were reported to host a large population of children with SAM.

In order to obtain a weighted sample, 4% of households were randomly selected using SPSS-generated random numbers from the districts’ Water, Sanitation and Hygiene survey registry. Households with no under-five children were replaced by new households using the randomly selected household list.

From August 2011 to January 2012, data collection was carried out by female nurses who were trained in the standardized use of anthropometric measurements. Data collectors were organized in teams and each had one assistant while taking anthropometry measurements. Home-visits were used to collect information on households, caregivers and children’s characteristics.

### 2.3. Measures

#### 2.3.1. Anthropometry

The weight of the children was measured to the nearest 0.1 kg using the United Nations International Children’s Emergency Fund (UNICEF) electronic scale. MUAC was taken using the WHO-recommended MUAC tape and procedure, and recumbent length and height were measured to the nearest 0.1 cm using UNICEF’s recommended model wooden board, as per the WHO protocol [22,23]. Interviewers were trained in anthropometric measurement techniques and each measurement was standardized by being taken twice and validated by calculating technical error of measurement (TEM). The WHO Anthro software (WHO, Geneva, Switzerland) was used to convert weight, height and age data into *Z*-scores using the 2006 WHO Growth Standards [24]. Wasting was defined as WHZ < −2 SD or MUAC < 125 mm. Severe wasting was defined as WHZ < −3 SD or MUAC < 115 mm [22]. Children with oedema (*n* = 33) and missing information on oedema (*n* = 13) were excluded from analyses as WHZ is heavily influenced by the weight of fluid retained in the body and can obscure low WHZ [6]. Anthropometric observations of WHZ (*n* = 42) and MUAC *Z*-score MUACZ (*n* = 13) outside the range −5 to +5 *Z*-scores were considered to be outliers and were excluded from the analyses [22].

#### 2.3.2. Demographics and Socio-Economic Status (SES)

Socio-demographic information on households, caregivers and children, such as age, marital status, education and occupation were collected. To describe the household’s economic status, the following socioeconomic indicators were used: housing construction materials of the dwelling in which the family resides, availability of electric power, source of drinking water, toilet facility, and size of owned and cultivated land at the time of interview. Furthermore, a household with a source of drinking water from a pubic/stand pipe, tube/protected well, or protected spring and rainwater were labelled as ‘improved’ [25]. If household’s toilet facility was a ventilated improved pit latrine or a pit latrine with a slab, it was also labelled as ‘improved’ [25].

#### 2.3.3. Statistical Analyses

Descriptive statistics were used to describe the age and sex distribution of the children. We created binary outcome variables for the diagnosis of severe wasting using MUAC and WHZ as described in the methods section. Proportions and 95% confidence intervals were computed to allow comparisons between indicators. The ages of the children were dichotomized to correspond to categories usually used in field nutrition programmes: young (age < 24 months), and older children (age ≥ 24 months). The kappa coefficient (κ) was calculated to assess the degree of agreement between MUAC and WHZ. The strength of agreement of the κ values was categorized as follows: κ ≤ 0.20, poor; 0.21 ≤ κ ≤ 0.40, fair; 0.41 ≤ κ ≤ 0.60, moderate; 0.61 ≤ κ ≤ 0.80, good; and 0.81 ≤ κ ≤ 1.00, excellent [26,27,28,29]. For the kappa coefficient analysis, we defined severe wasting based on the two anthropometric indicators as described earlier. Data were analysed using SPSS 20 for Windows statistical software package (International Business Machines Corporation, New York, NY, USA).

### 2.4. Ethics

The institutional ethical review board of Addis Continental Institute of Public Health (ACIPH) granted ethical clearance (ACIPH/IRB/002/2011) for the study and the project was reviewed by the regional ethical review board in Uppsala, Sweden. Permission to conduct the study was also obtained from regional and district health offices in Ethiopia. The Helsinki Declaration was followed when conducting the study. Mothers or primary caregivers were informed about the survey procedures and verbal consent was sought prior to interview.

## 3. Results

**Figure 1:**
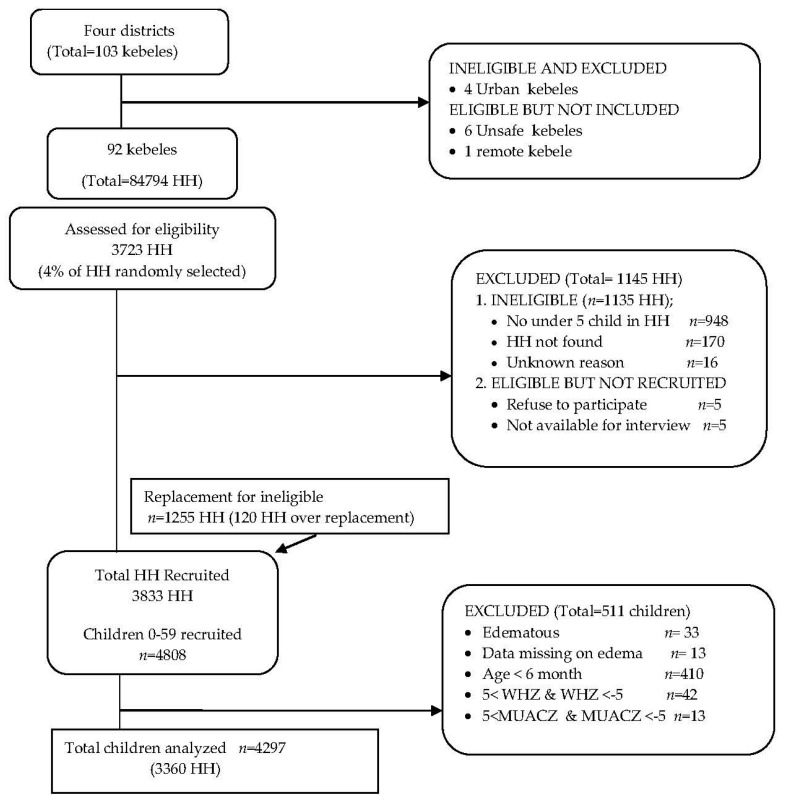
Participants flow chart (HH: household).

From the four selected districts, all of the rural kebeles (*n* = 99) were to be included in the survey but six had to be excluded due to security issues and one due to remoteness. Thus, 92 kebeles were included and randomly selected households were assessed for eligibility. We excluded ineligible households such as those with no under-five children (*n* = 948), households not found (*n* = 170) and those excluded for unknown reasons (*n* = 16); and those that were eligible but who refused or were not available for interview (*n* = 10). We replaced ineligible households with new ones (*n* = 1255). A total of 3833 households, in which 4808 under-five children resided, were recruited to participate in the study. In this paper, we excluded 511 children who did not fulfil the inclusion criteria: children who were under 6 months of age (*n* = 410), edematous (*n* = 33), missing data on edema (*n* = 13), and those with implausible *Z*-score values (*n* = 55). We compared the latter two groups of children with those included in the study, and found no significant difference in the age, sex or caregivers’ and household characteristics of the children. Thus, a total of 4297 children were included in our analyses (Figure 1).

The background characteristics of the households, caregivers and children are shown in Table 1. Household members typically lived in huts with wood and mud walls, earth floors with no electricity or improved toilet facility, and owned an average of 0.19 hectares of farming land. Mothers/caregivers were 31 years old on average, 88% were married, and 60% were farmers. The mean age of the children was 33 months and 51% were boys.

The prevalence, age and sex distribution of wasting among 6–59 month-old children depended on the indicator that was used (Table 2). MUAC categorized more children as wasted (10.5%, 95% CI: 9.6%, 11.4%) compared with WHZ (5.4%, 95% CI: 4.8%, 6.1%). For definition of severe wasting, MUAC categorized a larger proportion of girls compared with WHZ (Table 2). Similarly, MUAC categorized a larger proportion of young children (6–23 months) as severely wasted (3.9%, 95% CI: 3.0%, 5.1%) than when WHZ was used (2.0%, 95% CI: 1.4%, 2.9%).

MUAC and WHZ identified different populations of severely wasted children with only partial overlap (Table 3). We found no significant difference in other caregiver’s, household and child characteristics between the two groups of severely wasted children. Overall, 12/51 (23.5%) boys, 4/45 (8.9%) girls, 14/68 (20.6%) young children and 2/28 (14.3%) older children fulfilled both MUAC and WHZ criteria for severe wasting (Table 3). The degree of agreement as measured by kappa coefficient (κ), between anthropometric indicators of severe wasting, differed depending on the sex and age of the children (Table 3). The boys’ kappa coefficient was 0.37 and the girls’ was 0.15. Likewise, the degree of agreement was examined for each age group, and the young children’s kappa coefficient was 0.32 and the older children’s was 0.13.

## 4. Key Messages

MUAC and WHZ have fair agreement (κ = 0.28) in diagnosing SAM in children.

MUAC and WHZ have poor agreement in diagnosing SAM in girls (κ = 0.15) and older children (κ = 0.13).

## 5. Discussion

The two anthropometric indicators of severe wasting did not identify the same population of children as SAM. MUAC categorized a larger proportion of girls and young children as severely wasted compared with WHZ. For definition of severe wasting, there was fair agreement between MUAC and WHZ in boys and young children but poor agreement in girls and older children.

We excluded seven rural kebeles that were not accessible due to remoteness and safety issues, which could reflect a lower socio-economic environment. However, it is unlikely that this would influence our evaluation of the agreement between the indicators, as age and sex distribution of the children who participated in our study from all four districts were comparable. Although the presence of bilateral pitting edema is an independent indicator of SAM [13], it is known to introduce measurement bias in weight-based indicators [6]. Thus, we excluded edematous children from the analyses as it was deemed inappropriate for the comparison of WHZ and MUAC.

In the present study, MUAC categorized a larger proportion of children as wasted and identified children that differed according to age and sex as severely wasted compared with WHZ. Despite a considerable overlap in case definition by MUAC and WHZ, these indicators are also shown to categorize a different population of children as wasted [14,15,30,31,32,33]. Further, a study on agrarian and nomadic Ethiopian populations revealed that WHZ is substantially influenced by leg length [34]. While this suggests that the use of WHZ in SAM identification could overestimate wasting in populations with longer legs, MUAC may also overestimate SAM in children as it uses a fixed cutoff for all ages and both sexes. Thus, interpretations made based on anthropometric measurements may differ in different settings.

We found a larger proportion of girls categorized as severely wasted when using MUAC compared with WHZ. Others also reported similar findings [15,16]. As the growth reference values and MUAC are lower in girls [30,35], a single cut-off to define acute malnutrition may result in over diagnosis of the condition in girls compared with indicators that are standardized for age and sex [15,36]. While our results show the prevalence of wasting in boys to be higher than girls when WHZ was used, there was no sex-difference when MUAC was used. Findings from the nutrition rehabilitative programme using the WHO sex-specific WHZ table for programme admissions also revealed a higher proportion in the enrollment of boys [37], while programmes in community settings using MUAC as admission criteria found no sex-difference in programme admissions [38]. As girls are physically smaller than boys [35], a single cut-off to define severe wasting may result in overestimating the proportion of SAM in girls or under-estimating the proportion of SAM in boys. Thus, when making comparison between groups of children, it is important to consider age and sex composition during analyses.

Basic quality requirements of diagnostic methods include agreement of the methods [39]. Examining how well the methods agree involves measuring the extent to which the results of different test methods agree, and not merely the association or correlation of the results. In such a context, an agreement test should be used. Cohen’s kappa coefficient (κ) remains the most commonly used measure [27,39], mainly because it represents the chance-corrected proportional agreement for categorical variables [26,29]. The kappa statistic is dependent on the number of categories of response in that it tends to be relatively high when there are only two categories. The kappa is also affected by prevalence, much like predictive values are affected by the prevalence and, thus, it may not be appropriate to compare the kappa measurements between different studies [26,28]. However, it can provide more information than a simple calculation of the raw proportion of agreement [28]. Hence, in our study, kappa analysis determined the potential agreement between MUAC and WHZ in identifying SAM in children.

The strength of agreement between the anthropometric indicators of severe wasting was fair for young children and poor for older children. As MUAC increases with increasing age [9,30,34], a common cutoff value is likely to categorize more young children as wasted than older ones. Similarly, as WHZ and the WHZ-based definition of acute malnutrition have been shown to be associated with younger age and body shape of children [34], it may identify more young children as severely wasted than older children. Our findings are also consistent with the results of the above studies. However, the difficulties of taking accurate weight and height measurements compared to MUAC are well documented [7,40]. Furthermore, WHZ is shown to be largely influenced by leg length, which is not related to the nutritional status of the child, which may also decrease its ability to identify high-risk children [34]. Thus, considering MUAC’s simplicity [4,6], it appears to have an advantage in the early identification of malnourished children. Although this could raise an issue of increased programme volume when MUAC is used, there is actually a potential gain in terms of the efficient use of health workers’ time, avoiding hospitalization and its high health care expenditure [38].

Before making a decision regarding the use of WHZ and/or MUAC for admission to nutritional rehabilitative programmes, it is important to understand the indicators’ ability to predict mortality and whether this is related to age and sex. Younger children are at higher risk of mortality and are affected earlier in the wasting process before the onset of medical complications [38]. Research shows that MUAC is more sensitive at high specificity levels than WHZ in predicting mortality among children [9,10,15,20]. Further, MUAC has been shown to predict mortality independent of age [10,11,15,41], while the prediction of mortality using WHZ varied with age [42]. In addition, there was no difference found in a study comparing MUAC and sex-specific MUAC standardized for age (MUAC *Z*-score) in relation to predicting mortality in children under three years of age [36]. The same study reported that MUAC had made a similar prediction for mortality in boys and girls within 90 days [36], implying the usefulness of MUAC to identify children at high risk of dying. Similarly, in our study, the strength of the agreement did not improve and our results remained the same when MUACZ was used (result not shown), suggesting that the use of MUAC and MUACZ in identifying SAM in children is comparable. In order to reduce mortality, identifying which children would benefit most from treatment has been suggested to be more important for programmes providing care for SAM children [43]. Thus, the present analysis should be viewed as a step to clarify which anthropometric indicator could be more effective in identifying children at risk of dying and responds better to treatment.

A strength of the study was the measures taken to ensure good quality data were collected. The WHO-recommended MUAC tape and the UNICEF-recommended weight and height boards were used to assure quality of measurements. We conducted training and promoted the standardization of anthropometric measurements for all enumerators to minimize measurement errors and ensure the validity and precision of measurements in the fieldwork. Our interpretation of the results mostly apply to the study area and extrapolation of these results may require evaluation across various contexts.

## 6. Conclusions

The agreement between MUAC and WHZ to diagnose severe wasting among children aged 6–59 months varied according to age and sex. The poor agreement within different groups of children questions the indicators’ performance to predict risk of mortality. This may have important programmatic implications for community management of severe acute malnutrition. More research is needed on the response to treatment and the prediction of mortality using different anthropometric measurements in relation to the age and sex of children.

## Figures and Tables

**Table 1:** Household, maternal/caregiver and child characteristics of participants in rural south Ethiopia.

Characteristics	*n* or Mean ^a^	(%) or SD ^b^
*Household*		
Quality of housing		
Iron sheet roof (*n* = 3359)	1372	(40.8)
Earth floor (*n* = 3358)	3296	(98.2)
Wood and mud wall (*n* = 3359)	3131	(93.2)
Improved drinking water (*n* = 3358)	2711	(80.7)
Improved sanitation facility (*n* = 3332)	247	(6.5)
Electricity (*n* = 3358)	33	(1.0)
Owned land size (hectares) (*n* = 3360)	0.19 ^a^	0.17 ^b^
*Maternal/caregiver*		
Age (year) (*n* = 3319)	31 ^a^	8.6 ^b^
15–24	367	(10.9)
25–34	1811	(53.9)
35–44	818	(24.3)
≥45	323	(9.6)
Married (*n* = 3334)	2954	(87.9)
Education (*n* = 3352)		
No formal school	2121	(63.1)
Primary (Grade 1–8)	1164	(34.6)
Secondary/above	67	(2.0)
Occupation (*n* = 3354)		
Agriculture/farmer	1997	(59.5)
Petty trade/own skilled work	965	(28.7)
No occupation	303	(9.0)
*Child* (*n* = 4297)		
Age (months)	33 ^a^	15.9 ^b^
6–11	571	(13.3)
12–23	812	(18.9)
24–35	657	(15.3)
36–47	1215	(28.3)
48–59	1042	(24.2)
Male sex	2187	(50.9)

^a^ Values are presented as mean and ^b^ standard deviation.

**Table 2:** Prevalence of wasting as measured by weight-for-height *Z*-score (WHZ) and mid-upper arm circumference (MUAC) among 6–59 month children in rural south Ethiopia.

		Wasting				Moderate Wasting				Severe Wasting			
		WHZ < −2 SD ^∞^		MUAC < 125 mm		WHZ ≥ −3 SD and <−2 SD *^∞^*		MUAC ≥ 115 mm and <125 mm		WHZ < −3 SD *^∞^*		MUAC < 115 mm	
Age (Months)		%	(95% CI)	%	(95% CI)	%	(95% CI)	%	(95% CI)	%	(95% CI)	%	(95% CI)
6–23	Combined (*n* = 1383)	9.8	(8.3, 11.5)	20.8	(18.7, 23.1)	7.8	(6.5, 9.8)	16.9	(15.0, 19.0)	2.0	(1.4, 2.9)	3.9	(3.0, 5.1)
	Boys (*n* = 740)	11.6	(9.4, 14.2)	19.1	(16.3, 22.1)	8.9	(7.0, 11.3)	15.7	(13.2, 18.6)	2.7	(1.7, 4.2)	3.4	(2.2, 5.0)
	Girls (*n* = 643)	7.8	(5.9, 10.2)	22.9	(19.7, 26.3)	6.5	(4.8, 8.8)	18.4	(15.5, 21.6)	1.2	(0.6, 2.5)	4.5	(3.1, 6.5)
24–59	Combined (*n* = 2914)	3.3	(2.7, 4.0)	5.6	(4.8, 6.5)	2.7	(2.2, 3.4)	5.1	(4.3, 6.0)	0.5	(0.3, 0.9)	0.5	(0.3, 0.8)
	Boys (*n* = 1447)	3.8	(2.9, 5.0)	4.7	(3.7, 6.0)	3.1	(2.3, 4.2)	4.1	(3.2, 5.3)	0.7	(0.4, 1.3)	0.6	(0.3, 1.1)
	Girls (*n* = 1467)	2.8	(2.0, 3.8)	6.4	(5.2, 7.8)	2.4	(1.7, 3.3)	6.0	(4.9, 7.4)	0.4	(0.2, 0.9)	0.4	(0.2, 0.9)
6–59	Combined (*n* = 4297)	5.4	(4.8, 6.1)	10.5	(9.6, 11.4)	4.4	(3.8, 5.0)	8.9	(8.1, 9.8)	1.0	(0.8, 1.4)	1.6	(1.2, 2.0)
	Boys (*n* = 2187)	6.4	(5.5, 7.6)	9.6	(8.4, 10.9)	5.1	(4.2, 6.1)	8.0	(7.0, 9.3)	1.4	(0.9, 2.0)	1.5	(1.1, 2.1)
	Girls (*n* = 2110)	4.3	(3.5, 5.3)	11.4	(10.1, 12.9)	3.6	(2.9, 4.6)	9.8	(8.5, 11.1)	0.7	(0.4, 1.1)	1.7	(1.2, 2.3)

^∞^ Nutritional status (6–59 months) determined using WHO reference.

**Table 3:** Agreement between MUAC and WHZ to diagnose severe acute malnutrition by sex and age.

	WHZ < −3	−3 ≤ WHZ < −2	−2 ≤ WHZ	*Total*
**All Children**				
MUAC < 115	16	28	24	*68*
115 ≤ MUAC < 125	11	77	294	*382*
125 ≤ MUAC	17	83	3747	*3847*
*Total*	44	188	4065	*4297*
^†^ κ = 0.28				
Boys				
MUAC < 115	12	15	6	*33*
115 ≤ MUAC < 125	8	44	124	*176*
125 ≤ MUAC	10	52	1916	*1978*
*Total*	*30*	*111*	*2046*	*2187*
^†^ κ = 0.37				
Girls				
MUAC < 115	4	13	18	*35*
115 ≤ MUAC < 125	3	33	170	*206*
125 ≤ MUAC	7	31	1831	*1869*
*Total*	*14*	*77*	*2019*	*2110*
^†^ κ = 0.15				
<24 months				
MUAC < 115	14	21	19	*54*
115 ≤ MUAC < 125	6	50	178	*234*
125 ≤ MUAC	8	37	1050	*1095*
*Total*	*28*	*108*	*1228*	*1383*
^†^ κ = 0.32				
≥24 months				
MUAC < 115	2	7	5	*14*
115 ≤ MUAC < 125	5	27	116	*148*
125 ≤ MUAC	9	46	2697	*2752*
*Total*	*16*	*80*	*2818*	*2914*
^†^ κ = 0.13				

^†^ κ is calculated for severe wasting in 2 × 2 tables (MUAC < 115 vs. WHZ < −3) and numbers in cells are reported.
